# Factors Affecting Social Media Users’ Emotions Regarding Food Safety Issues: Content Analysis of a Debate among Chinese Weibo Users on Genetically Modified Food Security

**DOI:** 10.3390/healthcare9020113

**Published:** 2021-01-21

**Authors:** Hao Xiong, Shangbin Lv

**Affiliations:** School of Journalism and Communication, Wuhan University, Wuhan 430072, China; skystar816@whu.edu.cn

**Keywords:** social media, emotions, food safety, genetically modified foods, factors, content analysis

## Abstract

Social media is gradually building an online information environment regarding health. This environment is filled with many types of users’ emotions regarding food safety, especially negative emotions that can easily cause panic or anger among the population. However, the mechanisms of how it affects users’ emotions have not been fully studied. Therefore, from the perspective of communication and social psychology, this study uses the content analysis method to analyze factors affecting social media users’ emotions regarding food safety issues. In total, 371 tweet samples of genetically modified food security in Sina Weibo (similar to Twitter) were encoded, measured, and analyzed. The major findings are as follows: (1) Tweet account type, tweet topic, and emotion object were all significantly related to emotion type. Tweet depth and objectivity were both positively affected by emotion type, and objectivity had a greater impact. (2) Account type, tweet topic, and emotion object were all significantly related to emotion intensity. When the depths were the same, emotion intensity became stronger with the decrease in objectivity. (3) Account type, tweet topic, emotion object, and emotion type were all significantly related to a user’s emotion communication capacity. Tweet depth, objectivity, and user’s emotion intensity were positively correlated with emotion communication capacity. Positive emotions had stronger communication capacities than negative ones, which is not consistent with previous studies. These findings help us to understand both theoretically and practically the changes and dissemination of user’s emotions in a food safety and health information environment.

## 1. Introduction

Food safety is becoming increasingly important in daily life and has always been a global concern. As the most populous developing country, the issue of food safety in China is very complex and is closely related to social stability and government authority. In recent years, China has entered an important transition stage that has met the needs of people for food, but food safety and public health incidents often occur. For example, food safety incidents such as the “melamine milk powder” and “Sudan red duck eggs” incidents have occurred in China and have posed great challenges to consumers’ psychological endurance and confidence in the food safety environment [1]. Even in America, Europe, and other developed countries, many people cannot fully trust the existing food supply chain [2]. Public concern about food safety and health is a global problem.

The promotion and risk of genetically modified food (GMF or GMO) in the human living environment are also receiving considerable attention in relation to food safety and public health. Academia and society as a whole have participated in a long-term debate about consumer acceptance of GMF around the world [3]. The scientific debate on the safety of GMF is complex. Consumers may never be able to receive a definite answer on the basis of their trust in information communicators and the media [4,5]. Scientific research on GMF has found that they are no more dangerous than traditional foods [6,7], but many countries still have a large number of public control measures on them [5,8,9]. The topic of GMF security is often a source of debate on social media. In the view of many netizens, GMF is regarded as a major threat to biodiversity, the environment, and public health [10,11,12], and thus, they often display irrational negative emotions, such as panic, anxiety, or anger, regarding GMF [13,14]. It is worth noting that even though GMF may play an important role in reducing world hunger, panic remains among a large number of residents regarding the safety risks of GMF [15].

In the early days of the GMF debate in China, although Greenpeace was actively arousing public doubts regarding GMF, the public was not affected as a whole [16]. Household surveys in 2002 and 2003 showed that people’s knowledge of biotechnology was generally low, but they had a positive attitude towards GMF [17]. This also led to health communication on GMF to be focused on the provision of various forms of genetically modified (GM) knowledge in the following years [18]. In 2008, China launched a project related to genetically modified breeding technology, but soon after, a number of GMF security events caused a negative shift in the public attitude towards GMF [16]. In particular, after the Ministry of Agriculture issued safety certificates for two kinds of genetically modified rice and one kind of genetically modified corn in 2009, the attitude of the Chinese public towards GMF took a sharp negative turn [19]. Some scholars believe that the reason for this is the outbreak of the Sanlu milk powder safety incident in 2008, which aroused widespread concern among consumers regarding the safety of food technology [20,21,22]. At that time, the dissemination of social information was dominated by mass communication and consequentially, an environment with limited information and lack of communication caused people to generally accept the information reported by the news media. Due to the limited cognitive ability of GM at that time, the public formed a preconceived misunderstanding of a technology that they were not familiar with and were more inclined to pay more attention to negative news [16]. With limited information and vague risk awareness, the public subjectively constructed the risk perception of GM and generally adopted a refusal attitude toward GMF [23,24]. Therefore, risk information regarding GMF to public health or the environment became a hypothetical belief and a source of stereotyping and mistrust towards GM biological experts.

Since 2010, social media has been playing an increasingly important role in global health and environmental communication, and has been gradually building a new information environment that has become an important channel for people to obtain GMF information in addition to the traditional mainstream news media [25]. Many GMF security incidents have triggered an increase in negative public opinions on social media in China, for example Cui Yongyuan (a famous Chinese TV host) released a documentary about the investigation of GMF in the United States in March 2014 [26], three genetically modified safety certificates were approved and renewed by the Chinese government in January 2015 [27], and a suspected phenomenon of excessive planting of genetically modified corn in the northeast of China in January 2016 occurred [28]. Social media user emotions were not only reflected in the mistrust of the regulatory capacity of relevant government departments and the refusal of information provided by GM experts, but also in their resistance to GM experts as representatives of vested interests [29]. When resisting scientific conclusions, some users began to irrationally stigmatize GMF and GM technology, and the rumors of a “conspiracy theory” occupied the hegemony of discourse [30]. As this kind of public opinion is difficult to control and easily arouses negative emotions, rumors spread rapidly, thereby causing panic among the online population. Over the years, under the circumstances of fierce controversy regarding GMF security in China, it has become difficult to change the perception and risk perception of genetically modified food, even if public knowledge of biotechnology has improved [31]. This situation is inconsistent with the conclusion that there was a positive correlation between public biological knowledge and GMF acceptance in the research of some Western scholars [32,33,34]. Peoples’ attitudes towards GMF security had gone beyond the scope of scientific knowledge and are influenced by many other factors, such as emotion, trust, and social culture.

Based on Habermas’ public domain theory, in recent years, some scholars in the field of science communication have stated that the dissemination of health science knowledge should be very different from the governmental position of traditional science popularization and the position of the scientific community [35]. Instead, it should adopt the citizen position of equality and interaction in knowledge communication [36]. However, due to the intensification of emotions on both sides of the GMF security debate on social media, it is difficult to carry out effective dialogue and communication. It is difficult to reach a consensus on the diversity of subjects in the field of social media [37]. The consensus here is not the ultimate consensus in the philosophical sense, but the consensus form of social representation [38]. Moscovici defines social representation as “a knowledge system composed of various preconceptions, images, and values that has its own cultural meaning and continue to exist independent of individual experience” [39]. The function of representation is to conventionalize objects, people, or events and place them in a familiar category context [40]. Thus, he put forward the social representations theory (SRT), which explains how individuals deal with and build shared meaning, and how people share the meaning of building [41]. SRT plays an important role in helping people to analyze their understanding of scientific and technological risks and objects [14]. From the perspective of SRT, GMF is generally anchored on laboratory-made, unnatural, or immoral aspects. The public’s classification and naming of unfamiliar technology products reflect their specific emotions regarding GMF, whether it is positive or negative.

In the era of social media, the social representation of GMF security is formed under the continuous stimulation of various external information, which comes not only from the media, but also from the subjects themselves in the social situation [37]. People form a variety of social representations through personal experience, interpersonal interaction, and media influence [42]. It is difficult to change the public view once the social representation mechanism has been formed [14]. This form of social representation is characterized by not only the views of social members, but also their emotional experiences. Discussions on social media can be regarded as the external projection of social representation and the debate process reflects the interaction and dialogue between different groups [43]. In this kind of interaction, emotions are easily perceived and emotional expressions can easily stimulate emotional responses. A tweet, as a form of social representation, is not only the carrier of social culture, but also a metaphor of the internal psychological mechanism [44]. Concealed behind this are the emotions, psychological world, and cultural value of social media users [45]. When people encounter health information on social media, they do not often stop to think about it carefully but rather quickly compare it with a previously formed criteria to make decisions [46]. Emotional factors are represented in the cognitive framework that enables people to respond quickly [47] and provides people with a quick way to make judgments. People are more likely to invoke emotions in order to understand GMF security than to mobilize their own knowledge to think [18]. Based on these theoretical frameworks, this study focuses on social media tweets and user’s emotional representations, which play an important role in food safety communication.

Emotion plays a more important role than knowledge in influencing people’s risk perception of controversial food safety issues [17]. Lazarus defined emotion as “organized cognitive-motivational-relational configurations whose status changes with changes in the person-environment relationships as this is perceived and evaluated” [48]. Emotion is a type of psychological activity produced by individuals together with cognition and consciousness. Social media gives users opportunities to express their views on personal issues and provides its users with opportunities to build emotional bonds with others based on shared experiences or attitudes [49]. Such sharing is objective in nature and usually reflects the certain emotional state and emotion intensity of the individual [50]. A user’s emotion intensity in a tweet represents the emotional strength of each tweet as it spreads. Like any other social activity, online interaction evokes user’s emotion and fosters emotional style [51]. 

Emotional representation promotes the continuous expansion of the social experience to a certain extent, forming a collective cognition that transcends individual members and exists independently in society for a certain period of time [52]. Social media is very important for the dissemination of information and emotions, and this makes emotion as communicative as information. Emotions are fundamentally social and contagious [53,54]. When people read emotional content, they naturally want to talk, share, and react because doing so enables them to manage their emotions through social interaction [55] and return to an emotional balance [56]. Social media, where many people build relationships and easily interact, is an ideal place to share emotions. However, pessimists point that emotional catharsis, distortion of facts, and rude remarks are widespread on social media [57]. As the contents of social media can influence individuals’ knowledge, attitudes, and behavior, the official government accounts or ordinary users publishing food safety information can both trigger the emotions of others [58]. In addition, some scholars have found that news media companies reporting food safety scandals through their social media accounts can amplify consumer anxiety [59]. The communication space constructed by social media causes people with similar thoughts to generally gather together and emotion factors tend to dominate their interaction [60]. The publisher of a tweet is not only the subject of the emotion, but also the communicator of the emotion. The type of account for posting tweets may have a certain impact on the emotions of users in the tweets [54,58]. Based on the evidence, we hypothesize the following:

**Hypotheses** **1** **(H1a).** *The account type of the tweet is significantly related to the user’s emotion type in the tweet regarding food safety issues.*

**Hypotheses** **1** **(H1b).** *The account type of the tweet is significantly related to the user’s emotion intensity in the tweet regarding food safety issues.*

**Hypotheses** **1** **(H1c).** *The account type of the tweet is significantly related to the user’s emotion communication capacity in the tweet regarding food safety issues.*

The types of emotion are often different depending on the topics of the tweets. Different groups have different social representations of the same food safety event [61]. On Chinese social media platforms, user dialogues on food safety issues are usually intense and emotional, and a certain degree of public criticism of government policies and officials is allowed, which is also an important part of triggering negative emotion [62]. Since food safety is an environmental health issue that is almost beyond the control of the public, emotional reactions related to companies or institutions that fail to maintain food safety are relatively strong [63]. The stronger they believe that the relevant industry or government agency is responsible for the crisis discussed, the more negative their emotional response to these entities [64]. The public’s response to any food safety crisis is particularly strong. This negative emotion is often caused by anger toward weak government supervision or enterprise fraud, and it spreads rapidly on social media [58]. Based on the evidence, we hypothesize the following:

**Hypotheses** **2** **(H2a).** *The topic of the tweet is significantly related to the user’s emotion type in the tweet regarding food safety issues.*

**Hypotheses** **2** **(H2b).** *The topic of the tweet is significantly related to the user’s emotion intensity in the tweet regarding food safety issues.*

**Hypotheses** **2** **(H2c).** *The topic of the tweet is significantly related to the user’s emotion communication capacity in the tweet regarding food safety issues.*

Public awareness of food safety is usually attributed to the results of natural incidents or human intervention [65]. There is a logical connection between awareness of a food safety crisis and the negative emotions of those responsible for the crisis [66]. When a food safety incident occurs, social media users are likely to direct their emotions towards the government or related companies. This attribution of food safety responsibility may trigger public anger towards those individuals or organizations creating risks [65]. Based on the evidence, we hypothesize the following:

**Hypotheses** **3** **(H3a).** *The object of the user’s emotion in the tweet is significantly related to the user’s emotion type regarding food safety issues.*

**Hypotheses** **3** **(H3b).** *The object of the user’s emotion in the tweet is significantly related to the user’s emotion intensity regarding food safety issues.*

**Hypotheses** **3** **(H3c).** *The object of the user’s emotion in the tweet is significantly related to the user’s emotion communication capacity regarding food safety issues.*

Social media is the main source of misinformation regarding food safety, which has become a serious problem in China. People who are misinformed are increasingly spreading rumors about food safety on social media [58]. Since understanding food safety information requires a certain degree of professional knowledge, short texts cannot provide clear explanations and often cause communication problems. A study of more than 12,000 news articles published on Twitter in 2018 also found that rumors spread farther, faster, deeper, and wider than the truth because they were more novel and can stimulate emotional reactions, such as fear and disgust [67]. The depth of the content of a tweet is the degree to which its content penetrates into the essence of things and is thoroughly analyzed. It may affect a user’s emotion to a certain extent. Based on the evidence, we hypothesize the following:

**Hypotheses** **4** **(H4a).** *The depth of tweet content positively affects the user’s emotion type in the tweet regarding food safety issues.*

**Hypotheses** **4** **(H4b).** *The depth of tweet content is positively correlated with the user’s emotion intensity in the tweet regarding food safety issues.*

**Hypotheses** **4** **(H4c).** *The depth of tweet content is positively correlated with the user’s emotion communication capacity in the tweet regarding food safety issues.*

The low threshold and anonymity of information dissemination on social media promote the viral spread of fake news and rumors on the Internet [58]. As the Internet and social media have become major sources of information, an increasing number of users have become the victims of fake news. A report in 2016 showed that more than 60% of Chinese adult internet users were often exposed to online rumors, and social security and food safety were the two main types [68]. Some scholars believe that emotion plays an important role in the effect of misinformation [64]. Some others believe that misinformation only triggers negative emotions among people who have a high degree of trust in food safety [69]. Therefore, the objectivity of tweet content may affect user’s emotion. Based on the evidence, we hypothesize the following:

**Hypotheses** **5** **(H5a).** *The objectivity of tweet content is positively affected by the user’s emotion type in the tweet regarding food safety issues.*

**Hypotheses** **5** **(H5b).** *The objectivity of tweet content is negatively correlated with the user’s emotion intensity in the tweet regarding food safety issues.*

**Hypotheses** **5** **(H5c).** *The objectivity of tweet content is positively correlated with the user’s emotion communication capacity in the tweet regarding food safety issues.*

As a platform for communicating health issues, social media has been critically examined in relation to its accuracy of information, trustworthiness, and source credibility [70]. Negative information attracts people’s attention, intensifies their exposure to negative incidents that may cause feelings of pressure, and triggers group emotions among people [62]. Negative emotions mediated the level of misinformation and information transmission [58]. Compared with neutral emotions, negative emotions that may cause disgust, fear, or happiness are more likely to spread from person to person, and they are spread more widely through social media [70,71,72]. Negative emotions tend to cause people to pay more attention, while there is less focus on positive emotions [73,74]. Although a study shows that articles with positive emotions have fewer user reviews [67], some studies have found that positive articles generate more user sharing behaviors and have greater dissemination influence [75]. Based on the evidence, we hypothesize the following:

**Hypotheses** **6** **(H6a).** *The user’s emotion type in a tweet is significantly related to their emotion communication capacity in the tweet regarding food safety issues.*

**Hypotheses** **6** **(H6b).** *The user’s emotion intensity in a tweet is positively correlated with their emotion communication capacity in the tweet regarding food safety issues.*

A brief review of the literature shows that scholars have a certain foundation to understanding the characteristics of a user’s emotion communication on social media. However, due to the interdisciplinary characteristics, current research has generated very little data on internal factors that affect user emotions regarding food safety issues. In the food safety information environment, the reasons and mechanisms behind social media message dissemination affecting the emotional response of users have not been thoroughly studied. As user emotions are affected by many environmental factors, this study combined communication variables with psychology variables to analyze the factors affecting the emotions of social media users regarding food safety issues.

## 2. Materials and Methods 

From the perspective of communication, content analysis is a research method to objectively, systematically, and quantitatively describe the content of communication [76]. We used the content analysis method to encode and analyze tweets on food safety issues in Chinese social media and to explore the factors affecting user’s emotions, such as the account type, topic, depth, objectivity, and object of the tweets.

### 2.1. Study Sample

Sina Weibo has been the leader in the development of domestic social media platforms since it was established on 14 August 2009. Weibo had 523 million monthly active users and 229 million daily active users in October 2020 [77]. As Sina Weibo ranks first in the country in terms of the number of monthly active users, daily active users, comprehensive influence, etc., it was selected as the research platform in this study. As a channel for Chinese people to discuss social problems, Weibo has increasing influence. The issue related to GMF security has continuously been a topic of great interest on Weibo. Using Weibo as the sample pool and utilizing the “advanced search” function, the search time was fixed from 1 January 2018 to 31 December 2019. A total of 8400 tweets were obtained by searching for original tweets (non-forwarded tweets) on Weibo with “genetically modified food” as keywords. Then some tweets were extracted by the method of isometric sampling for research and analysis, and a total of 420 samples were obtained. In order to ensure that all samples were related to GM food safety issues, 49 duplicate and invalid samples were manually excluded, and 371 valid samples remained.

### 2.2. Measures

On the basis of previous research [78,79], 8 research variables were determined, and combined with the definition of variables in the abovementioned literature and assumptions. The coding content and definitions for all of the variables are shown in Table 1.

The tweet’s account type is the publisher type that each tweet belongs to. It was divided into the following items: (1) Government agency; (2) news media; (3) GMF enterprise (corporate that produces or sells GMF); (4) industry expert (scholar in the food or relevant industry and official accounts of industry associations); (5) opinion leader (celebrities); and (6) ordinary user with a code from 1 to 6 in turn.

A tweet topic refers to the main content that users want to express through the information posted on social media [80]. It was divided into the following items: (1) Regulation and law; (2) health news; (3) enterprise behavior (advertise, marketing products, and not labeling GM-related information on products); (4) expert opinion; (5) health risks (risks to personal or public health); (6) scientific cognition (or scientific literacy); and (7) phenomenon thinking (user’s self-examination of food safety and the health environment) with a code from 1 to 7 in turn.

Emotion object in the tweet is the object of the user’s emotion in the tweet, such as a person or an organization. It was divided into the following items: (1) Government agency; (2) news media; (3) GMF enterprise; (4) industry expert; (5) opinion leader; and (6) ordinary user with a code from 1 to 6 in turn.

Tweet depth is the detailed level of opinions or information given in the content of the tweet. The number of words in tweets can reflect the formal depth of the article from a certain extent. According to this, the levels of tweet depth were determined via a 5-point scale, and were divided into the following items: (1) Ranges from 0 to 30 Chinese characters; (2) from 31 to 60; (3) from 61 to 90; (4) from 91 to 120; and (5) from 121 to 140.

Tweet objectivity is the degree to which its content information is accurate and can truly reflect objective facts. The level of it ranged from 0 to 5, with 5 indicating the highest level of objectivity.

User’s emotion type in the tweet is the user’s emotional tendency in the process of emotional transmission [74], which was divided into 3 items: (1) Positive, (2) neutral, and (3) negative.

User’s emotion intensity in the tweet is the emotional strength of each tweet as it spreads. The level of it was determined via a 5-point scale, ranging from very weak (1) to very strong (5).

User’s emotion communication capacity in a tweet is the ability of each tweet to influence the thoughts and actions of others in the process of emotional transmission. According to the total number of retweets, comments, and likes, the level of it was determined via a 5-point scale and was divided into the following items: (1) The number is 0; (2) ranges from 1 to 20; (3) from 21 to 40; (4) from 41 to 60; and (5) greater than 60.

The content of each tweet was statistically analyzed as a unit on the basis of the above 8 indicators, and 3 encoders were selected for evaluation. A total of 2986 judgments were required. In order to ensure the reliability of content analysis, the 3 encoders jointly developed a content analysis framework and exchanged their understanding of the connotation of coding for many times. The reading and content coding of all of the samples were completed independently by encoder A and encoder B. After two rounds of independent evaluation, the number of coding results was 2432. According to the formula of the average mutual consent degree:

(1) $$K=\frac{2M}{N1+N2}$$

where N1 is the number of categories analyzed by encoder A, and N2 is the number of categories analyzed by encoder B. The average mutual consent degree (K = 0.81) was calculated to measure the degree of mutual agreement between the two coders. Then according to the formula of the content analysis method’s reliability:

(2) $$R=\frac{n\times K}{1+(n-1)\times K}$$

where *n* is the number of encoders. The reliability R was 0.90. For the items with inconsistent evaluation results, the coding was finally determined with further consultation with encoder C.

### 2.3. Data Analysis

To explore the relationship between the above variables, chi-square tests, analyses of variance, Student–Newman–Keuls tests, and multilevel linear regression analyses were performed with IBM SPSS Statistics 20.0. The first step was to analyze the characteristics and influencing factors of user’s emotion type in tweets. The second step was to analyze the characteristics and influencing factors of user’s emotion intensity. The third step was analysis of the characteristics and influencing factors of user’s emotion communication capacity. Finally, the relationships among emotion type, intensity, and communication capacity were analyzed.

## 3. Results

Through the descriptive statistical analysis of 371 tweet samples (Table 2), it was found that the main types of user’s emotion regarding food safety issues were negative emotions (62%), followed by positive emotions (20.8%), with neutral emotions account for the lowest proportion (17.2%). Negative emotions accounted for a high proportion, and most of them were expressed as criticisms, fears, doubts, and anger. The majority of the user’s emotion communication capacity was level 1 (49.6%), followed by level 2 (38.5%), while tweets with a level 5 influence only accounted for 5.1%. This may be because tweets were mostly posted by ordinary users, thereby causing the overall influence to not be so high.

### 3.1. Factors Affecting Users’ Emotion Type

Chi-square tests were conducted to ascertain the interrelationships between account type, tweet topic, emotion object, and the user’s emotion type. The results showed that the account type (*X^2^* = 122.420, *p* = 0.000), tweet topic (*X^2^* = 481.015, *p* = 0.000), and emotion object (*X^2^* = 250.840, *p* = 0.000) were all significantly related to user’s emotion type. Therefore, H1a, H2a, and H3a are supported.

In order to further understand this correlation, we made cross-analyses of the above variables. The results are shown in Table 3.

Among the positive emotions, industry experts accounted for the largest proportion (53.1%). They mostly called on the public to scientifically understand the risks of GMF and the knowledge related to GM technology with the aim of guiding user’s emotions towards a positive view. Topics about scientific cognition (78.8%) accounted for the highest proportion. This reflects the notion that someone advocated social media users to understand GMF rationally, appealed to them to identify rumors, and cultivated personal scientific literacy. Positive emotions that take the ordinary user (85.7%) as the emotion object were the most dominant form of emotion. Since government agencies, media, and industry experts were trying to dredge the negative emotions of ordinary people, this caused their emotions to be calmer.

Among the neutral emotions, tweets from government agencies (43.3%) and news media (26.7%) represented the majority. The accounts of government agencies used serious language when publishing information, did not declare its position on whether GMF is safe or not, and tried to be as impartial. News media accounts paid attention to objectivity and truthfulness and tried their best to avoid emotional judgments thus, both government agencies and news media accounts were generally neutral. Topics about phenomenon thinking (50.0%) accounted for the highest proportion, and the emotions were more rational, and mainly reflected thoughts about the governance of food safety problems and the construction of a healthy communication environment. Neutral emotions that take the ordinary user (40.6%) as an emotion object were the most dominant form of emotion.

Among the negative emotions, opinion leaders accounted for the largest proportion (77.4%). Celebrities who lack professional knowledge usually questioned, satirized, and criticized the safety of GMF. Topics about regulation and law (52.6%) accounted for the highest proportion, followed by topics about expert opinion (20.9%). The objects of negative emotion were mainly government agencies (54.8%), industry experts (21.7%), and GMF enterprises (17.8%). 

As tweet depth, tweet objectivity, and type of emotion were all serial variables, correlation analysis was conducted on them. The results show that with the strengthening of depth (*r* = −0.236, *p* = 0.000) and objectivity (*r* = −0.717, *p* = 0.000), user’s emotions changed from negative to positive. Tweet depth and objectivity were both positively affected by a user’s emotion type. Therefore, H4a and H5a are supported. In addition, it also showed that objectivity had a greater impact than depth.

### 3.2. Factors Affecting Users’ Emotion Intensity

Analyses of variance were conducted to ascertain the interrelationships between account type, tweet topic, emotion object, and user’s emotion intensity. The results showed that the account type (*F* = 15.204, *p* = 0.000), tweet topic (*F* = 26.308, *p* = 0.000), and emotion object (*F* = 18.856, *p* = 0.000) were all significantly related to the user’s emotion intensity. Therefore, H1b, H2b, and H3b are supported.

In order to further understand this correlation, Student–Newman–Keuls tests were conducted to identify the mean values of items that were significantly different from each other, and then to group them according to the user’s emotion intensity. The results are shown in Table 4.

The results show that there were no significant differences among the items in groups A1 (*p* = 0.378), A2 (*p* = 0.712), B1 (*p* = 1.000), B2 (*p* = 0.146), B3 (*p* = 0.205), C1 (*p* = 0.976), and C2 (*p* = 0.155). This means that when the account type was a government agency (*M* = 2.25), news media (*M* = 2.53), or industry expert (*M* = 2.73), the emotion intensity was usually weak. On the contrary, when it was a GMF enterprise (*M* = 3.50), ordinary user (*M* = 3.66) or opinion leader (*M* = 3.78) the emotion intensity was usually strong. 

When the tweet topic was phenomenon thinking (*M* = 2.14), the emotion intensity was usually weak; when it was scientific cognition (*M* = 3.05), enterprise behavior (*M* = 3.50), or health risks (*M* = 3.52) the emotion intensity was usually of medium strength; and when it was regulation and law (*M* = 3.87), health news (*M* = 4.00), or expert opinion (*M* = 4.04), the emotion intensity was usually strong. 

When the emotion object was an opinion leader (*M* = 2.82) or ordinary user (*M* = 2.83), the emotion intensity was usually weak. On the contrary, when it was a GMF enterprise (*M* = 3.46), news media (*M* = 3.63), government agency (*M* = 3.80), or industry expert (*M* = 4.04), the emotion intensity was usually strong.

Multilevel linear regression analyses were conducted by taking the user’s emotion intensities as dependent variables with depth and objectivity as independent variables in turn. The results showed that there was no significant correlation between the tweet depth (*p* = 0.578 > 0.05) and emotion intensity. Therefore, H4b is not supported. When the influence of depth was controlled, the second layer of linear regression analysis showed that tweet objectivity (*β* = −0.648, *p* = 0.000) negatively correlated with the user’s emotion intensity in the tweet. Therefore, H5b is supported. When the depth was the same, the user’s emotion intensity became stronger with the decrease in tweet objectivity.

### 3.3. Factors Affecting Users’ Emotion Communication Capacity

Analyses of variance were conducted to ascertain the interrelationships between account type, tweet topic, emotion object, and the user’s emotion communication capacity. The results showed that the account type (*F* = 29.005, *p* = 0.000), tweet topic (*F* = 5.386, *p* = 0.000), and emotion object (*F* = 4.003, *p* = 0.002) were all significantly related to the user’s emotion communication capacity. Therefore, H1c, H2c, and H3c are supported.

In order to further understand this correlation, we conducted a Student–Newman–Keuls test on the above variables to identify the mean values of items that were significantly different from each other, and then to group them according to the emotion communication capacity. The results are shown in Table 5.

The results show that there were no significant differences among the items in group A1 (*p* = 0.137), A2 (*p* = 0.614), B1 (*p* = 0.178), B2 (*p* = 0.171), C1 (*p* = 1.000), C2 (*p* = 0.317), and C3 (*p* = 0.243). This means that when the account type was an ordinary user (*M* = 1.46), GMF enterprise (*M* = 2.00), government agency (*M* = 2.00), or industry expert (*M* = 2.18), the emotion communication capacity was usually weak. Conversely, when it was news media (*M* = 2.89) or an opinion leader (*M* = 3.06), the emotion communication capacity was usually strong.

When the tweet topic regarded health news (*M* = 4.00), regulation and law (*M* = 3.87), phenomenon thinking (*M* = 1.00), or enterprise behavior (*M* = 3.50) the emotion communication capacity was usually weak, however when it was health risks (*M* = 3.52), expert opinion (*M* = 4.04), or scientific cognition (*M* = 3.05), the emotion communication capacity was usually strong.

When the emotion object was a government agency (*M* = 1.32), the emotion communication capacity was usually weak; when it was an GMF enterprise (*M* = 1.92), opinion leader (*M* = 2.03), or news media (*M* = 2.21), the emotion communication capacity was usually of medium strength; and when it was an ordinary user (*M* = 3.54) or industry expert (*M* = 3.76), the emotion communication capacity was usually strong.

Multilevel linear regression analyses were conducted by taking the user’s emotion communication capacities as dependent variables and objectivity and depth as independent variables in turn. In the first layer of linear regression analysis, the results show that tweet objectivity (*β* = 0.49, *p* = 0.000) positively correlated with the user’s emotion communication capacity. Therefore, H5c is supported. When the influence of objectivity was controlled, the second layer of linear regression analysis showed that tweet depth (*β* = 0.638, *p* = 0.000) positively correlated with the user’s emotion communication capacity. Therefore, H4c is supported. When the tweet objectivity was the same, the user’s emotion communication capacity became stronger with the increase in tweet depth.

### 3.4. Correlation between User’s Emotion Type, Intensity, and Communication Capacity

A multilevel linear regression analysis was made by taking the user’s emotion communication capacities as dependent variables and the type and intensity as independent variables in turn. In the first layer of linear regression analysis, the results show that emotion type (*β* = −0.181, *p* = 0.000) significantly correlated with the user’s emotion communication capacity. Therefore, H6a is supported. When the emotion type was controlled, the second layer of linear regression analysis showed that emotion intensity (*β* = 0.269, *p* = 0.000) positively correlated with the user’s emotion communication capacity. Therefore, H6b is supported. When the type of emotion was the same, the user’s emotion communication capacity became stronger with the increase in intensity.

## 4. Discussion

### 4.1. Users’ Emotion Type Regarding Food Safety Issues

From the perspective of the communicator, the user’s emotion types presented by tweets regarding food safety issues varied according to the account type. Opinion leaders often showed negative emotions and played an important role in interpersonal communication on social media. People were more likely to change their attitude when they received information from the people they admired [81]. This was the main reason why the overall types of user’s emotion regarding food safety issues tended to be negative. Conversely, government agencies, news media, and industry experts mostly tried to dispel the negative feelings of the public. The remarks made by government agencies and news media were mainly neutral, calling on the public to think calmly and treat food safety risks rationally. Most of the tweets posted by industry experts showed positive emotions, and tried to guide user’s emotions to a positive view by introducing GMF technologies.

From the perspective of communication content, the user’s emotion type changed with tweet topics. On the one hand, when tweet topics were related to regulation and law people were most likely to make comments with negative emotions based on the notion that the current managements are not strict enough. When the tweet content discourse involved the views of industry experts, the public were more likely to express negative opinions and most of them doubted the judgment of experts and accused experts of being immoral. On the other hand, with the decrease in objectivity and depth of the tweet content, the users’ emotion type was more likely to change negatively, and objectivity has a greater impact on users’ emotion type than depth. Tweets with negative emotions were mostly the irrational catharsis of ordinary users and could not stand scrutiny. The information in these tweets was incomplete or left blank and was mostly composed of rumors or fake news. On the contrary, tweets with positive emotions tended to be more reasonable and dealt with food safety problems from a scientific perspective in order to spread health knowledge or dispel public negative emotions, and the content of their dissemination was more objective and profound.

From the perspective of the communication object, the users’ emotion types regarding food safety issues varied according to the emotion object. When the object of emotion was a government agency, it was more likely to cause negative comments, which is consistent with previous studies [82,83]. When the emotion object was an opinion leader, positive comments were more easily produced, with some opinion leaders being regarded by users as heroic figures who dare to think and act. It can be seen observed that opinion leaders were more likely to resonate with user’s emotions [84].

### 4.2. Users’ Emotion Intensity Regarding Food Safety Issues

From the perspective of the communicator, the user’s emotion intensity presented by tweets on food safety issues varied according to the account type. The emotions of government agencies, news media, and industry experts were relatively weak. This may be because they shoulder greater social responsibility, try to actively intervene to dispel negative public emotions with relative calmness [83], and appear more calm when expressing their opinions. On the contrary, the related enterprises, ordinary users, and opinion leaders were directly affected by food safety incidents therefore, their emotional intensities were strong. The related enterprises that carry out public relations in crises were eager to declare their position and therefore, their emotion was firm. Opinion leaders mostly questioned and criticized the supervision work of food safety risks, and their emotion intensity was strong. Under the influence of the emotions of opinion leaders and stimulated by their own irrational needs, ordinary user’s emotions became intense [81].

From the perspective of communication content, the users’ emotion intensity presented by tweets changed with a tweet’s topics. When topics involved regulations, health news, and industry expert opinions, the emotion intensity was strong. When content involved the reflection of public health, the emotion intensity was weak. Meanwhile, in the same content depth, the emotion intensity decreased with the improvement in the objectivity of a tweet. With the improvement in objectivity, the users’ interpretation of food and health knowledge were more accurate, expression in tweets was more rational, and emotion intensity was reduced.

From the perspective of the communication object, the users’ emotion intensity regarding food safety issues varied according to the emotion object. When the users’ emotion object was government agencies (*n* = 154) or industry experts (*n* = 54), it was easy to generate strong emotions. Both government agencies and industry experts tried to dispel public emotions and guide public opinion in a more rational and calm manner, but the effects of emotional guidance were not ideal. On the contrary, fewer emotions were directed to opinion leaders (*n* = 11), and the emotion directed to opinion leaders was lower in intensity. Opinion leaders’ comments were more likely to arouse public emotion and be recognized by ordinary users.

### 4.3. Users’ Emotion Communication Capacity Regarding Food Safety Issues

From the perspective of the communicator, the users’ emotion communication capacity regarding food safety issues varied according to the account type. Industry experts have higher professional ability and scientific literacy, but it was difficult for them to spread scientific or health knowledge to reach more social media users, and it was also difficult for users to accept their judgement or form a certain concept. Opinion leaders did not perform as well as industry experts in professional knowledge and technology, and while the government and news media performed better than them in terms of bureaucracy and authority, they had a greater influence on the emotions of social media users [80]. This may be because opinion leaders have rich information resources, information channels, and a large number of fans, and have accumulated popularity through a long-term operation. As a result, they more credibility with social media users. Opinion leaders were considerably skillful in stimulating content or publishing blank content to attract a large number of fans. They expressed their views and analyzed problems from the side of the users, thereby avoiding one-way communication.

From the perspective of communication content, the users’ emotion communication capacity changed with the content of tweets. Firstly, the change in topics brought about a change in the users’ emotion communication capacity. The focus of the debate among users was mostly on whether GM technology is moral, whether the views of industry experts are credible, and whether GMF is healthy. The three topics have strong communication power on social media. Although the number of topics related to regulation and law (*n* = 133) was the highest, most of them were commented on by ordinary people, and the communication capacity was very limited. Secondly, the users’ emotion communication capacity can be enhanced with the improvement of tweet depth and objectivity. The more accurate the information about food safety given by users, the more they can truly reflect objective facts or phenomena, and the further they explore nature. In addition, the more likes, comments, and retweets they receive, the more influential their emotions will be.

From the perspective of the communication object, the users’ emotion communication capacity regarding food safety issues varied according to the emotion object. When the emotion objects were industry experts and ordinary users, the communication capacity was strong because these represent two opposite sides in the food safety debate. Experts called on the public to obtain a rational understanding of food safety issues and improve their scientific literacy. However, ordinary users questioned or opposed expert views.

In addition, the users’ emotion communication capacity varied according to the emotion type and intensity. Firstly, when the types were the same, stronger emotion intensity had better communication capacity. Secondly, the emotion communication capacity usually decreased as the emotion tendency changed from positive to negative. Previous studies have shown that positive emotions reduce people’s attention [62,63,80] and that disgust and fear spread more widely through social media [70,71,72], but the results of this study are not consistent with these notions. This is mainly because food safety is an issue that is close to people’s livelihood. The negative emotions were mainly posted by ordinary users and although they were large in number, the personal influence of ordinary users was normally lower than that of government agencies, news media, or industry experts. Therefore, the overall negative emotion communication capacity was limited.

### 4.4. A Social Representation Comparison between China and Western Countries

People are usually ambivalent about GMF in China, with emotional conflicts occurring on social media. On the one hand, some people hope that new technologies will increase grain production and improve people’s quality of life, while on the other hand, many people associate new technologies with the field of risk due to the uncertainty surrounding GMF. For ordinary persons, the emotional representation of food safety includes not only the judgment of the probability and consequences of food safety incidents but also the hope that relevant enterprises and regulatory authorities can actively take actions or measures to properly deal with food safety risks in order to reduce the incidence of food safety incidents in China. It is worth noting that the Chinese population presents unique emotional representations for different emotion objects. The negative emotions in response to industry experts or the government are usually more intense than those regarding companies involved in food safety accidents. “Facts” give way to “emotions”, and trust becomes a scarce resource in food safety issues. The spread of official words is often met with a “Tacitus trap”, which has been extended as a modern social phenomenon by Chinese scholars. When a government department or an organization loses its credibility, regardless of whether they are telling the truth or lying, the belief of the public will not be aroused and the institution in question will be regarded as telling lies and doing bad things [16,17]. Some experts who support GMF tried to carry out corrective explanations for related problems, which were exclusive in the sense of representation. Opinion leaders changed the authoritative discourse mode of “monologue style” to the interactive mode of “dialogue style” [10], which made it easier to gain public emotional recognition. In addition, GMF enterprises only equated the social representation of GMF with the representation of certain technology or technological products, which rejected the sociological and cultural interpretation of GMF and lacked a communication process that combines science and technology with Chinese culture.

GM technology originated in the United States, but the debate about GMF in the United States has not been fierce and people seldom form polarized emotions regarding this issue [85]. The United States usually avoids discussing GM, hoping to play down the complexity of GM issues and reduce public misgivings about GM, emphasizing the notion that GMF has advantages of high yield, fewer fertilizers, and high quality [86]. In terms of public psychological cognition, the risk of GMF does not cause too many negative emotions, especially among people with high academic qualifications [85]. American media pays less attentions to GMF and the public trust in the government’s GMF safety standards and regulations is relatively higher than that of the Chinese public [87].

However, social representation of GMF is considerably different in Europe. Even the attitudes of people with high academic qualifications towards GMF often show a form of polarization [88]. Although the EU (European Union) takes a cautious attitude towards GMF and actively promotes public participation, this has not stopped Europeans from questioning the safety of GMF. In 1996, when the United States approved the first batch of commercially grown GM crops and exported them to Europe, there was an outcry from environmental non-governmental organizations (NGO), and the social controversies around GM in Europe have escalated since then [88]. After entering the 21st century, Europeans continued to boycott GMF and a number of studies have found that in the news of some European countries, negative or skeptical content regarding GM has occupied the mainstream [89]. When the EU reviewed the relevant regulations on GM in 2003, a number of demonstrations against GM broke out in European countries [90]. Research showed that a considerable number of Europeans care about the risks of GMF and felt uneasy about the ability of regulators to protect consumers from GMF risks [91,92]. A public survey in Denmark found that GM crops, such as common industrial insect-resistant crops, have been highly demonized by opponents and described as a desecration of the natural order concluded by God [93]. In fact, from the view point of European culture, the GMF dispute is closer to a continuation of social struggles, such as protests against environmental pollution or the opposition to nuclear power.

### 4.5. Implications for the Management of the Food and Health Information Environment

How can users’ negative emotions and the communication capacity of food and health information be improved? How can an efficient, accurate, and rational food and health information environment be built? The research results provide us with some insights regarding these questions. Firstly, the official account of government agencies should change the overly serious discourse system, strengthen emotional communication with people, further improve the mechanism of food information disclosure, improve the efficiency of administrative supervision, and disclose relevant food information immediately. Secondly, when reporting food safety and health information, news media accounts should not only transmit relevant information or health knowledge but also improve the depth of the content, pay more attention to improving public scientific literacy, and exercise the educational function of media. Thirdly, experts can make full use of the advantages of new technology to transfer knowledge or express opinions in a more active way so as to avoid an excessively academic discourse system. Finally, the social media platform should pay more attention to fake health information expressed by opinion leaders who do not have professional heath knowledge, so as to quickly identify and dispel rumors in time to avoid further spread of negative emotions among users. Additionally, more opinion leaders with professional knowledge in the field of food and public health should be trained to spread accurate food safety information and dispel public negative emotions in a timely fashion in food safety incidents. If different representation subjects can communicate with each other in a more transparent and collaborative way, the emotional identity and resonance between them will be more stable, and health information will gain a more substantial meaning.

### 4.6. Limitations and Future Research

Our results have some limitations. We focused on specific groups, primarily users from Chinese social media. This choice had a certain regional nature and fails to provide possible uniform population and national conditions, thus limiting the possible deviations caused by unobserved heterogeneity. As this may limit the generality of the results, further research is needed to confirm them. Additionally, tweet depth was coded according to the number of words, which does not exactly reflect a detailed level of the opinions or information. Tweet objectivity and emotion intensity are too subjective to measure and other methods can be used for a cross-verification.

Adding additional research variable such as age, gender, educational level, or economic status, may help to better classify users and conduct more effective research on factors affecting emotions in the future.

## 5. Conclusions

This study analyzed the factors affecting social media users’ emotions regarding food safety issues in the cultural context of China. We found that tweet account type, tweet topic, and emotion object were all significantly related to the users’ emotion type, intensity, and communication capacity. Tweet depth and objectivity were both positively affected by a users’ emotion type, and objectivity had a greater impact. When the depths were the same, the users’ emotion intensity became stronger with a decrease in objectivity. Tweet depth, tweet objectivity, and users’ emotion intensity were positively correlated with emotion communication capacity. 

The social expression of GMF in China is an interactive and dynamic social process. It not only depends on the improved communication strategy but also on the changes in the users’ psychology and social culture. The negative emotions of social media users regarding GMF are not only a crisis of scientific cognition, but they are also influenced by local culture. Promoters of GMF try to characterize the product as relatively neutral information or genetic engineering technology. They attempt to regard it as a scientific representation, but it is closer to social representation. Therefore, it conceals the meaning production, value preference, and emotional resonance contained in the representation. These meanings and value preferences do not fully match the concept map of the public. As a result, there was a certain degree of confrontation between the scientific community and nonprofessional groups.

Few studies have focused on factors affecting users’ emotions regarding social media health communication. This study has made some theoretical contributions in relation to factors affecting peoples’ emotions and provides case samples from China. These findings help us to understand both theoretically and practically the changes and dissemination of user’s emotions regarding food safety and the health information environment. This study can facilitate the development of a clearer understanding of Chinese peoples’ attitudes and emotions regarding food safety issues. It also provides guidance on how to build a stable, safe, and interactive health information environment for public health management departments, food safety organizations, and health media. Using these findings, managers can take appropriate preventive or remedial measures to reduce the irrational negative emotions of social media users regarding food and health issues. 

## Figures and Tables

**Table 1:** Coding table for content analysis of tweets about genetically modified food (GMF) security.

Research Variables	Coding Content
Account type	(1) government department; (2) news media; (3) GMF enterprise; (4) industry expert; (5) opinion leader; (6) ordinary user
Tweet topic	(1) regulation and law; (2) health news; (3) enterprise behavior; (4) expert opinion; (5) health risks; (6) scientific cognition; (7) phenomenon thinking
Emotion object	(1) government department; (2) news media; (3) GMF enterprise; (4) industry expert; (5) opinion leader; (6) ordinary user
Tweet depth	(1) ranges from 0 to 30 Chinese characters; (2) from 31 to 60; (3) from 61 to 90; (4) from 91 to 120; (5) from 121 to 140.
Tweet objectivity	5-point scale, ranges from 0 (lowest level) to 5 (highest level)
Emotion type	(1) positive; (2) neutral; (3) negative
Emotion intensity	(1)very weak; (2)relatively weak; (3)medium; (4)relatively strong; (5)very strong
Emotion communication capacity	total number of retweets, comments and likes (1) is 0; (2) ranges from 1 to 20; (3) from 21 to 40; (4) from 41 to 60; (5) greater than 60

**Table 2:** Descriptive statistical analysis results (*n* = 371).

Variables	Items	*n*	%
Type of emotion	Positive	77	20.8
	Neutral	64	17.2
	Negative	230	62
Intensity of emotion	Level 1	10	2.7
	Level 2	51	13.7
	Level 3	119	32.1
	Level 4	128	34.5
	Level 5	63	17
Communication capacity of emotion	Level 1	184	49.6
	Level 2	143	38.5
	Level 3	17	4.6
	Level 4	8	2.2
	Level 5	19	5.1

**Table 3:** Cross-analyses of emotion type (*n* = 371).

Variables	Emotion Type		
	Positive	Neutral	Negative
Account type	industry expert (53.1%)	government agency (43.3%) news media (26.7%)	opinion leader (77.4%)
Tweet topic	scientific cognition (78.8%)	phenomenon thinking (50.0%)	regulation and law (52.6%) expert opinion (20.9%)
Emotion object	ordinary user (85.7%)	ordinary user (40.6%)	government agency (54.8%) industry expert (21.7%) GMF enterprises (17.8%)

**Table 4:** Student–Newman–Keuls (SNK) test results of emotion intensity.

Variables	Group	*p*	Items	Intensity
Account type	A1	0.378	government agency; news media; industry expert	weak
	A2	0.712	GMF enterprise; ordinary user; opinion leader	strong
Tweet topic	B1	1.000	phenomenon thinking	weak
	B2	0.146	scientific cognition; enterprise behavior; health risks	medium
	B3	0.205	regulation and law; health news; expert opinion	strong
Emotion object	C1	0.976	opinion leader; ordinary user	weak
	C2	0.155	GMF enterprise; news media; government agency; industry expert	strong

**Table 5:** SNK test results of emotion communication capacity.

Variables	Group	*p*	Content	Communication Capacity
Account type	A1	0.137	ordinary user; GMF enterprise; government agency; industry expert	weak
	A2	0.614	news media; opinion leader	strong
Tweet topic	B1	0.178	health news; regulation and law; phenomenon thinking; enterprise behavior	weak
	B2	0.171	health risks; expert opinion; scientific cognition	strong
Emotion object	C1	1.000	government agency	weak
	C2	0.317	GMF enterprise; opinion leader; news media	medium
	C3	0.243	ordinary user; industry expert	strong

## Data Availability

Data sharing not applicable.
